# Flexible Dual Fabry–Perot Filters Enabling Color Balanced Narrowband Emission Designed by Physics Guided Neural Inverse Optimization

**DOI:** 10.1002/nap2.70135

**Published:** 2026-05-13

**Authors:** Keun Soo Shin, Ki Won Jeong, Yun Seon Do

**Affiliations:** ^1^ School of Electronic and Electrical Engineering Kyungpook National University Daegu South Korea

**Keywords:** bi‐directional neural network, color purity, dual Fabry‐Perot interferometer, flexibility, next generation displays, thin film optics

## Abstract

Achieving high color purity through precise spectral control is a key optical requirement for advanced display systems. Here, we present a flexible dual Fabry–Perot interferometer (DFPI) consisting of two stacked Fabry–Perot cavities, where one generates multiple resonance peaks and the other precisely tunes their spectral alignment and intensity. The optical behavior of the DFPI was analytically modeled as the product of individual Fabry–Perot factors (FPFs) and experimentally verified on a PET substrate. Furthermore, a data‐driven bi‐directional neural network (Bi‐NN), integrating forward and inverse models was employed to improve design efficiency, providing an accurate mapping between structure and optical variables and mitigating the non‐uniqueness issue in inverse prediction. The optimized Bi‐NN predictions exhibited excellent agreement with the analytical formulation results. When integrated with a blue LED and quantum dot color conversion films, the fabricated DFPI film reduced the spectral full width at half maximum (FWHM) and expanded the color gamut by over 30% relative to sRGB gamut, while maintaining excellent spectral and color stability under repeated bending conditions. This integrated optical and AI design framework offers a promising pathway for realizing next generation flexible displays with high color purity and reproducibility.

## Graphical abstract

The dual Fabry–Perot interferometer (DFPI) with functionally separated cavities enables simultaneous RGB peak alignment and spectral rebalancing without subpixel patterning. Multi resonance generation and fine spectral tuning are independently controlled, enhancing the color purity. A physics‐guided bi‐directional neural network accelerates inverse structural design for robust and efficient photonic design.

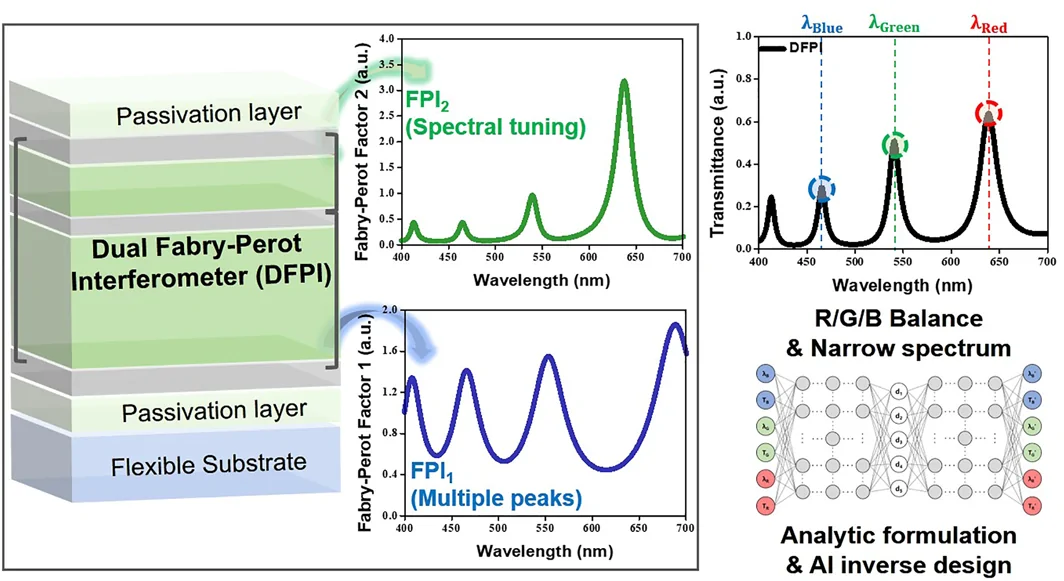

## 1 Introduction

The evolution of display technology has transcended a mere conduit for information, advancing toward providing a realistic and immersive user experience. The quality of visual experience is influenced by the following factors: resolution, which defines the image clarity [1]; contrast ratio, which indicates the difference between the darkest and brightest colors [2], and color reproduction, which represents color accuracy and range. Among these, color purity, a core parameter for color reproduction, is defined by the area of a triangle formed by the red, green and blue primary color coordinates in CIE‐1931 international standard color space [3, 4]. Despite significant progress, current international standards such as sRGB and Rec‐2020 are inherently limited in their capacity to reproduce the full spectrum of colors prevalent in nature, covering only 35.9% and 63.0% of the entire color gamut, respectively. This limitation highlights the critical need to enhance the color purity to achieve wide color gamut [5]. The importance of this endeavor is amplified in next‐generation applications, including micro‐LEDs and head‐up displays [6, 7], as well as emerging immersive technologies like augmented, virtual, and mixed reality [8, 9]. Advancements in color purity directly influence image clarity and accuracy, thereby profoundly impacting the user's perception of visual quality. Therefore, achieving high color purity is recognized as a critical requirement for elevating display performance, driving extensive and continuous research efforts in this domain.

High color purity can be realized through two primary methodologies. The first approach involves material‐level engineering to narrow the full width at half maximum (FWHM) of the emission spectrum. Organic systems utilize multiple‐resonance thermally activated delayed fluorescence (MR‐TADF) emitters to provide intrinsically narrowband spectra [10, 11]. On the inorganic system, perovskites enable precise band‐gap tuning via compositional and crystal structure control [12, 13], while quantum dots (QDs) exploit quantum confinement effect to control the emission wavelength. In particular, QDs offer a high photoluminescence quantum yield and wavelength tunability by controlling their particle size, and their solution processability facilitates large‐area fabrication [14, 15, 16]. However, QDs face challenges in achieving an ideal narrow bandwidth due to the use of toxic heavy metals such as cadmium (Cd) and lead (Pb), as well as spectral broadening caused by reabsorption and particle size distribution under fluorescent conditions [17]. To overcome these shortcomings, a complementary approach utilizes an optical structure that can selectively transmit or reflect specific wavelengths based on principles of resonance and interference, without altering the fundamental material properties. Notable examples of this strategy include Fabry–Perot interferometer (FPI) [18, 19, 20, 21] and distributed bragg reflector (DBR) [22, 23] based on multilayer thin films allowing precise wavelength control by tuning optical distance, the thickness and refractive index in each layer. In general imaging devices consisting of R, G, and B pixelized configuration, the thin film optical structure requires the different resonance conditions for R, G, and B wavelengths, requiring a complex patterning process for each sub‐pixels.

To overcome the constraints in the wavelength selectivity of the thin film structures, this paper proposes a dual Fabry–Perot interferometer (DFPI) structure comprising a cascaded two FPIs. The configuration consists of three reflective layers with two dielectric layers between them, enabling concurrent generation of distinct resonance peaks corresponding to R, G, and B wavelengths. The independent functionality of two FPIs provides multiple degrees of freedom for tuning the spectral position and intensity of each resonance peak. Consequently, yielding a transmission spectrum precisely tailored to the intended color range and thereby enabling the RGB color balance. A comprehensive analytical framework was established to quantitatively model overall transmission properties of the DFPI, drawing on the Fabry–Perot factor (FPF) of the combined structures. This created a foundation for the design that allowed for the quantitative assessment of the effects of variations in refractive index and layer thickness on the entire transmission spectrum.

Traditional optical design methods such as numerical analysis or electromagnetic (EM) simulations involve a substantial amount of trial and error to derive an optimal structure. As the complexity of the optical design increases, the required computational energy grows exponentially. Recently, photonic design with data‐driven learning utilizing artificial neural networks (ANNs) is actively underway [24, 25]. Once the dataset is acquired, learning can be accomplished, and with its outstanding prediction and computational energy, it can immediately replace EM simulation for optical structure design. In particular, an inverse neural network (INN), which forecasts structural variables from multiple optical characteristics, is fundamentally limited by the non‐uniqueness problem inherent in all inverse scattering problems. This issue arises because a single optical response can be generated by multiple, distinct structures, which is a primary factor hindering network convergence correctly during training. In this paper, we propose an inverse design model of DFPI as a rapid and exact design method. The basic approach is under a bi‐directional NN (Bi‐NN) that combines both INN and forward neural network (FNN) employed [26, 27, 28, 29]. This network effectively circumvents the one‐to‐ many mapping problem by feeding the INN's predictions through the pre‐trained FNN and comparing the resulting response with the original target response. The domain space was prepared by the extruded physical formula. With the optimized Bi‐NN model to the DFPI structure design, we could efficiently and accurately derive the thickness of each layer corresponding to the target R, G, and B peak wavelengths and their intensities in the transmission spectrum. Finally, the optimized DFPI was demonstrated experimentally in a flexible film type ensuring mechanical flexibility and stable optical characteristics under strain and deformable environments. This patterning‐free approach allows for direct integration with a wide range of optical devices without requiring additional processing steps, offering a pathway to improved color purity and an expanded range of applications without structural modifications to current display technologies.

## 2 Results

### 2.1 Background and Design Basis

We propose a DFPI structure fabricated on a PET substrate, to be utilized as a flexible optical film. As shown in Figure 1a, the structure consists of three silver (Ag) layers serving as reflectors, with tungsten trioxide (WO_3_) dielectric layers existing between them. Ag was selected due to its low refractive index in the visible region and high conductivity, minimizing optical absorption. To enhance the transmittance of light passing through the device, it is crucial to suppress surface plasmon (SP) coupling at the Ag interface. Thus, WO_3_ with a relative permittivity higher than that of Ag was chosen as the dielectric layer. Additional WO_3_ layers are placed above and below the DFPI structure to suppress SP coupling at Ag interfaces, thereby enhancing optical transmittance. Also, WO_3_ functions as a passivation layer, protecting the device from oxygen and moisture. Compared to direct deposition of Ag onto PET, introducing a bottom WO_3_ layer significantly improves adhesion, which is beneficial for uniform thin‐film formation.

**FIGURE 1:**
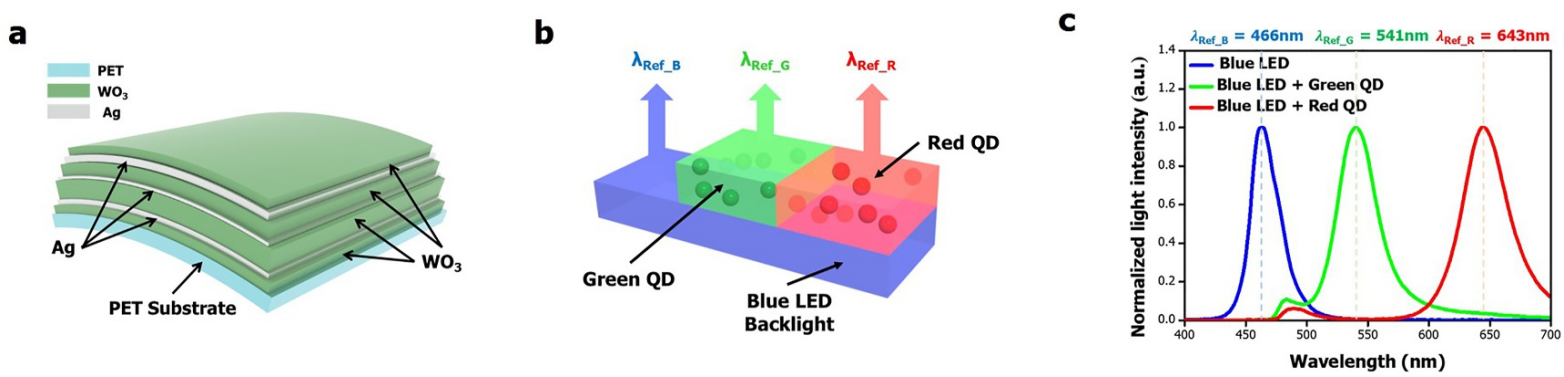
Schematic of the flexible dual Fabry–Perot interferometer (DFPI) structure and spectral characteristics of the reference light source. (a) Structural configuration of the designed flexible DFPI film. (b) Configuration of the reference light source consisting of a blue LED with green and red quantum dot (QD) color conversion layers. (c) Normalized emission spectra of the blue LED and green and red QDs with spectral peak wavelengths at 466, 541, and 643 nm, respectively.

In this paper, the DFPI structure was designed to achieve two optical performances. First, the transmission spectrum of DFPI should have both R, G, and B spectral peak wavelengths aligned with those of reference light. Second, the primary color balance was considered by adjusting the spectral intensities according to the actual amounts of B, G, and R in the reference light. For this experiment, reference lights were generated by combining a blue LED with green and red QD color conversion films, as shown in Figure 1b. The normalized emission spectra of reference lights are presented in Figure 1c. The blue LED backlight has a spectral peak wavelength (*λ*_ref_B_) at 466 nm. The photoluminescence (PL) spectral peaks of green and red QD films, serving as color conversion layers, were located at 541 nm (*λ*_ref_G_) and 643 nm (*λ*_ref_R_), respectively. The emission intensities measured at the peak wavelengths were 61,392 W/m^2^ for blue, 36,144 W/m^2^ for green, and 25,694 W/m^2^ for red. The spectrum's FWHM were 25.38, 36.49, and 43.66 nm for B, G, and R, respectively. Considering that the red region exhibited the lowest light intensity among the three primary colors, the DFPI structure was designed to achieve the highest transmittance in the red region.

### 2.2 Analytical Foundation for the DFPI Structural Design

An optimal DFPI structure was designed using the transfer matrix method. (Detailed information on transfer matrix and Fabry–Perot factor is provided in Supporting Information S1: S3 and S4) The optical and structural information required for DFPI design is illustrated in Figure 2a. In this study, the two Fabry–Perot interferometers are designated as FPI_1_ and FPI_2_. FPI_1_ (multiple peaks) is designed to generate multiple spectral peaks based on higher‐order resonance modes. FPI_2_ (spectral‐tuning) is applied to modulate the relative transmittance of each spectral region, thereby enabling fine‐tuning of color balance and purity.

**FIGURE 2:**
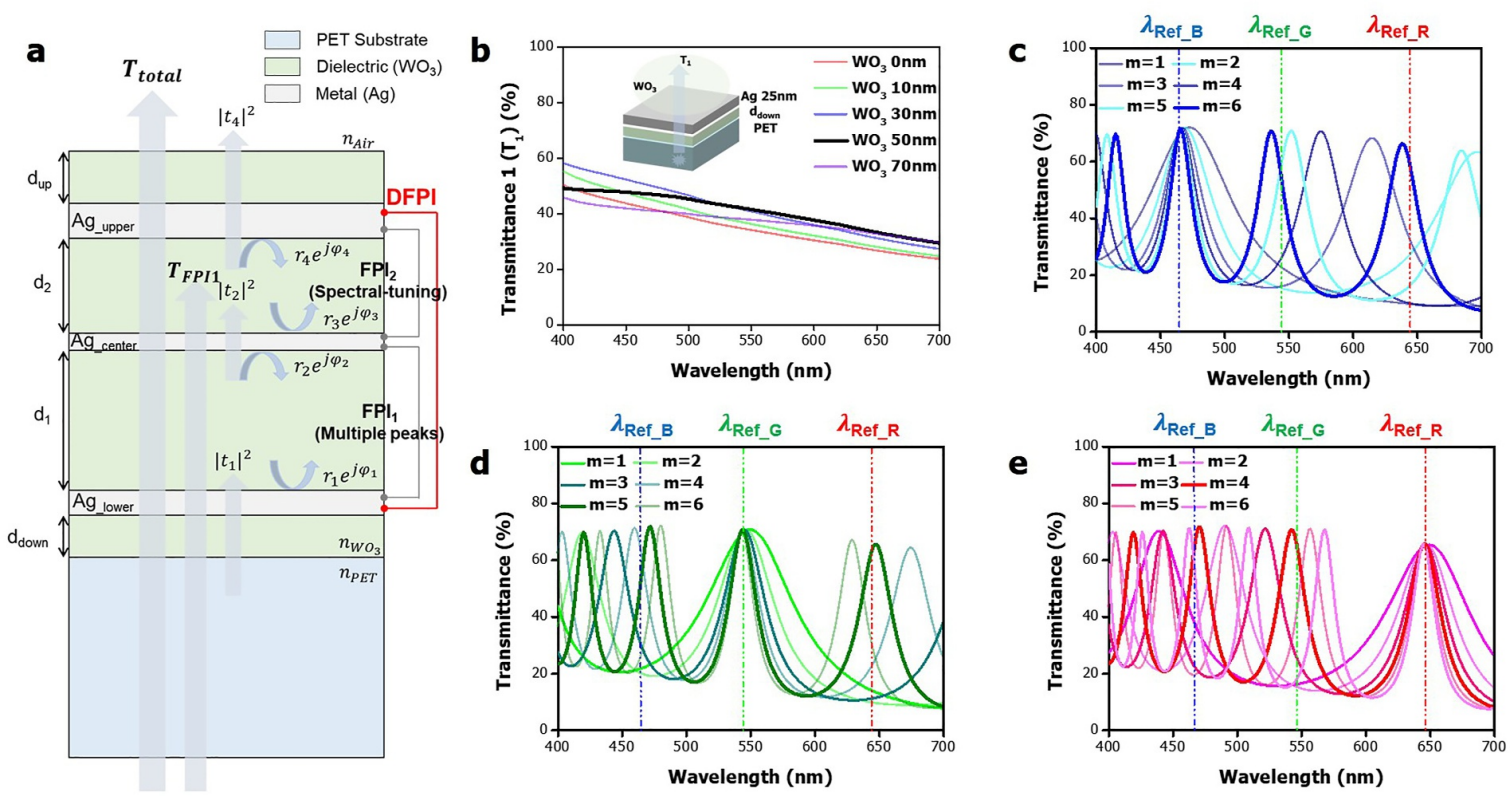
Higher‐order resonance mode analysis of FPI_1_. (a) Schematic of DFPI structure and definition of optical and structural parameters used in analytical model. (b) Transmittance (*T*_1_) variations as d_down_ changes. (c–e) Transmittance spectra designed to match *λ*_ref_B_ = 466 nm, *λ*_ref_G_ = 541 nm, and *λ*_ref_R_ = 643 nm, respectively.

The first transmittance ($T_{1}$) through the PET substrate, passivation layer, and lower Ag layer (Ag__lower_) to the dielectric layer of FPI_1_ is calculated by Equation (1).

(1) $$T_{1}=\frac{n_{{WO}_{3}}}{n_{PET}}\times {\left|t_{1}\right|}^{2}$$

Here, $n_{{WO}_{3}}$ and $n_{PET}$ denote the refractive indices of WO_3_ and PET, respectively, and $t_{1}$ represents the transmittance coefficient across these layers to the dielectric layers of FPI_1_. To evaluate the effect of WO_3_ on the PET substrate, Figure 2b shows the variation of $T_{1}$ with respect to the lower dielectric thickness (*d*_down_), ranging from 0 to 70 nm. When *d*_down_ was 50 nm, $T_{1}$ was the highest in the red spectral region and exhibited a uniform distribution across the visible region. Thus, the thicknesses of the thin film were set to 50 nm for WO_3_, 25 nm for Ag from below.

First, we verified whether a single FPI on the PET substrate could generate transmission peaks corresponding to *λ*_ref_B_, *λ*_ref_G_, and *λ*_ref_R_. Conventional FPI structures amplify optical resonance at wavelengths satisfying constructive interference conditions between two reflectors. By using higher‐order resonance modes, multiple transmission peaks can be generated based on specific resonance wavelengths.

The bottom layers of FPI_1_ consist of WO_3_ 50 nm and Ag 25 nm, while the top layer is composed of 10 nm of Ag. The Fabry–Perot factor 1 (FPF_1_) was obtained based on the reference wavelengths *λ*_ref_B_, *λ*_ref_G_ and *λ*_ref_R_, calculated based on FPI_1_ and the results for resonance order from first to sixth are shown in Supporting Information S1: S5, respectively.

(2) $${FPF}_{1}=\frac{{\left|t_{2}\right|}^{2}}{1+{R_{a}}^{2}-2R_{a}\;\cos\left({\Delta \varphi }_{FP1}\right)}$$

(3) $$R_{a}=|r_{1}r_{2}|e^{-2\kappa_{{\mathrm{WO}}_{3}}k_{0}d_{1}}$$

(4) $${\Delta \varphi }_{FP1}={-\phi }_{1}\;{-\;\phi }_{2}+2n_{{WO}_{3}}k_{0}d_{1}$$

The reflection coefficients at the lower and upper reflectors of FPI_1_ are denoted by $r_{1}$ and $r_{2}$, respectively, and their corresponding phases are represented by $\phi_{1}$ and $\phi_{2}$. The transmission coefficient $t_{2}$ describes light propagation from the dielectric layer of FPI_1_ through the center Ag (Ag__center_) into the dielectric layer of the FPI_2_. The refractive index of WO_3_ is expressed as a complex form, $n_{{WO}_{3}}-j\kappa_{{WO}_{3}}$, where $n_{{WO}_{3}}$ is the real part and $\kappa_{{WO}_{3}}$ is the imaginary part (extinction coefficient). The free‐space wavenumber *k*_0_ satisfies the relation *λ* = 2π/*k*_0_, where *λ* is the wavelength.

(5) $${\Delta \varphi }_{\mathrm{FP}1}=2m\pi$$

(6) $$d_{1}=\frac{\left(2m\pi +\phi_{1}+\phi_{2}\right)}{4n_{{\mathrm{WO}}_{3}}\pi }\lambda$$

$$T_{\mathrm{FP}I1}=T_{1}\times {\mathrm{FPF}}_{1}$$

(7) $$=\frac{n_{{WO}_{3}}}{n_{PET}}\frac{{\left|t_{1}\right|}^{2}{\left|t_{2}\right|}^{2}}{\left[1+{R_{a}}^{2}-2R_{a}\;\cos\left({\Delta \varphi }_{\mathrm{FP}1}\right)\right]}$$

The FPF_1_ was calculated using higher‐order resonance conditions, derived by rearranging Equation (4). The WO_3_ layer thickness *d*_1_ in FPI_1_ was determined using Equation (6) to satisfy the resonance wavelengths for *λ*_ref_B_, *λ*_ref_G_ and *λ*_ref_R_, where the resonance order number m starts from 0, where *m* = 0 corresponds to the first resonance mode. The transmission spectra of FPI_1_ designed to target *λ*_ref_B_, *λ*_ref_G_ and *λ*_ref_R_ are shown in Figure 2c–e, respectively. These spectra were calculated based on Equation (7). The corresponding resonance peak wavelengths obtained from the transmittance spectra of the FPI_1_ are denoted as *λ*_FPI_B_, *λ*_FPI_G_ and *λ*_FPI_R._ When the sixth‐order resonance mode (*m* = 5) is applied to match *λ*_ref_B,_ the resulting peaks are *λ*_FPI_G_ 536 nm and *λ*_FPI_R_ 638 nm, which differ by 5 nm from *λ*_ref_G_ 541 nm and *λ*_ref_R_ 643 nm, respectively. Similarly, when FPI_1_ is tuned to the green wavelength using fifth‐order resonance mode (*m* = 4), it yields *λ*_FPI_B_ 471 nm and *λ*_FPI_R_ 646 nm, with deviations of 5 and 3 nm, respectively. Likewise, matching *λ*_ref_R_ using fourth resonance mode (*m* = 3) yields *λ*_FPI_B_ 470 nm and *λ*_FPI_G_ 542 nm, which do not perfectly matched with *λ*_ref_B_ and *λ*_ref_G_.

These results indicate that FPI_1_ generates multiple resonance peaks through higher‐order resonance modes. However, achieving precise spectral coincidence with the three reference wavelengths (*λ*_ref_B_, *λ*_ref_G_ and *λ*_ref_R_) across the visible region remains challenging.

To achieve simultaneous alignment of *λ*_FPI_B_, *λ*_FPI_G_ and *λ*_FPI_R_ with the reference light peak wavelengths, an additional FPI structure FPI_2_ was introduced above the FPI_1_. A parameter study was conducted to evaluate the optical change of each layer in FPI_2_. For this analysis, the FPI_1_ was designed to include *λ*_ref_B_ in its transmission spectrum, consisting of WO_3_ and Ag layers at 50 nm, 25 nm, 635 nm, and 10 nm, respectively, on a PET substrate.

As shown in Figure 2a, the FPI_2_ was designed to include a top WO_3_ layer. The thickness of this layer (d_up_) was determined based on the variation of in fourth transmittance ($T_{4}$), defined as the transmission of light originating from the dielectric layer of the FPI_2_, passing through the 35 nm Ag and the WO_3_ layer, and proceeding to air. (Detailed information on $T_{4}$ are provided in Supporting Information S1: S6). The range of d_up_ was varied from 0 to 80 nm, and corresponding $T_{4}$ are plotted in Figure 3a. As mentioned in Figure 1c, a higher transmittance in the red region is desirable because of the relatively lower intensity of reference light. Therefore, d_up_ was chosen to be 60 nm, where the average red region transmittance is highest.

**FIGURE 3:**
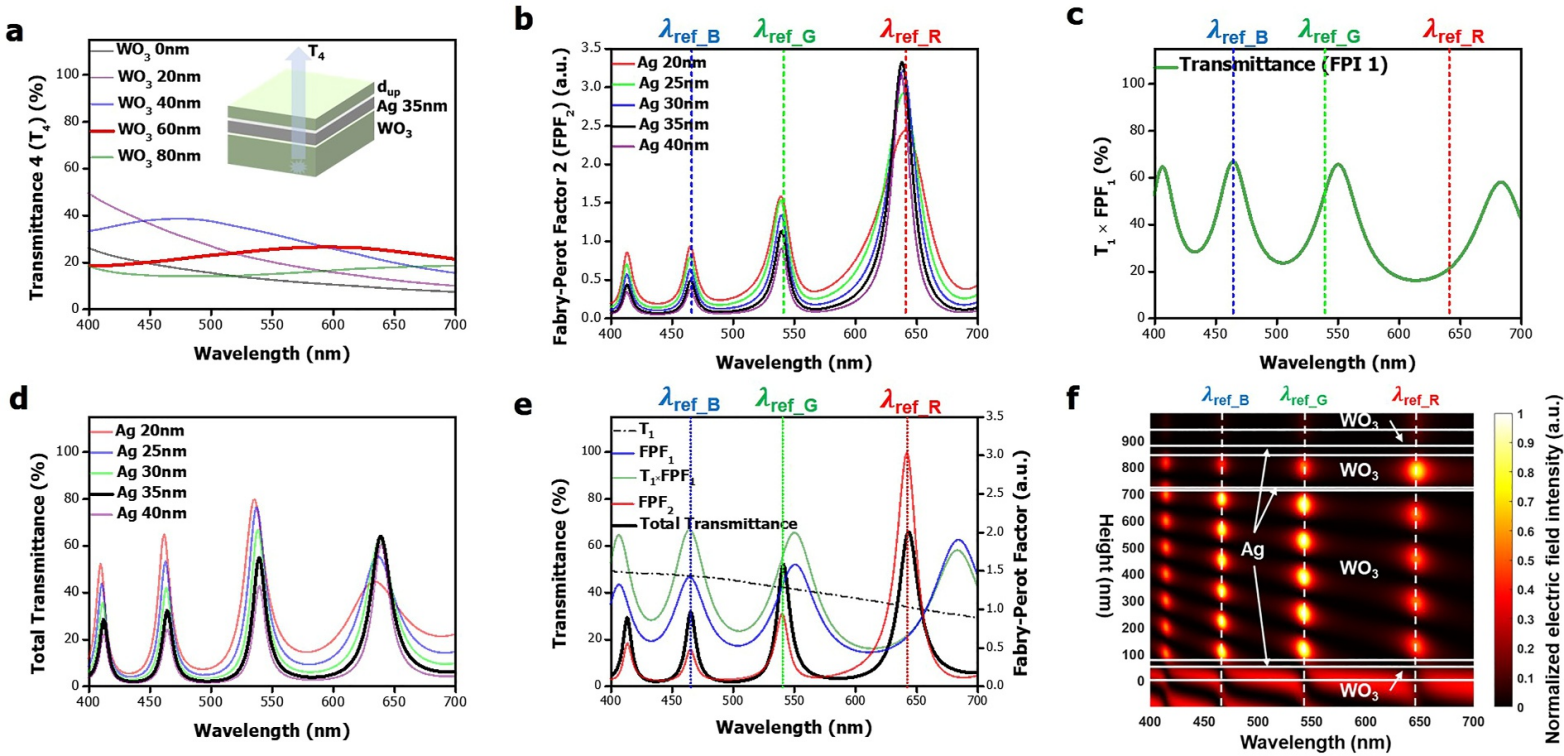
Integrated DFPI design with optical tuning and parametric optimization. (a) Transmittance (*T*_4_) variations as d_up_ changes. (b) Fabry–Perot factor (FPF_2_) graph of FPI_2_ designed to match *λ*_ref_R_ 643 nm. (c) Transmittance spectrum of FPI_1_ obtained from the product of *T*_1_ and FPF_1_. (d) Dependence of the overall DFPI transmittance on the Ag__upper_ thickness. (e) Schematic representation of the DFPI transmittance formulation derived from the step by step product of *T*_1_, FPF_1_, and FPF_2_. (f) Electric field distribution at the peak wavelengths of 466, 541, and 643 nm in the optimized DFPI cross‐section.

The resonance phenomenon between the upper Ag layer of the FPI_2_ (Ag__upper_) and underlying FPI_1_ was studied by varying Ag__upper_ thickness from 20 to 40 nm in 5 nm increments. The corresponding FPF_2_ of FPI_2_ designed for *λ*_ref_R_ is shown in Figure 3b and calculated using Equations ((8), (9), (10), (11), (12)).

(8) $${FPF}_{2}=\frac{n_{Air}}{n_{{WO}_{3}}}\frac{{\left|t_{4}\right|}^{2}}{\left[1+{R_{b}}^{2}-2R_{b}\;\cos\left({\Delta \varphi }_{\mathrm{FP}2}\right)\right]}$$

(9) $$R_{b}=|r_{3}r_{4}|e^{-2\kappa_{{\mathrm{WO}}_{3}}k_{0}d_{2}}$$

(10) $${\Delta \varphi }_{\mathrm{FP}2}={-\phi }_{3}\;{-\phi }_{4}+2n_{{WO}_{3}}k_{0}d_{2}$$

(11) $${\Delta \varphi }_{\mathrm{FP}2}=2m\pi$$

(12) $$d_{2}=\frac{\left(2m\pi +\phi_{3}+\phi_{4}\right)}{4n_{{\mathrm{WO}}_{3}}\pi }\lambda$$

The reflection coefficients at the lower and upper interfaces of the FPI_2_ are denoted by $r_{3}$ and $r_{4}$, and corresponding phase terms are represented by $\phi_{3}$ and $\phi_{4}$. At this point, $r_{3}$ assumes the light propagates from FPI_2_ downward through FPI_1_ to the PET substrate. The refractive index of air is denoted by $n_{Air}$ and $t_{4}$ is the transmittance coefficient of light from the dielectric layer of FPI_2_ through Ag__upper_ and top WO_3_ layer into air. The remaining optical variables follow the same definitions provided in the previous FPF formulation.

The resonance conditions specific to the FPI_2_ are given in Equations (11) and (12), and FPF_2_ was calculated using low‐order resonance modes of *m* = 1 for green and *m* = 0 for red. While varying the thickness of the upper Ag layer from 20 to 40 nm, the optimal thickness of *d*_2_ in the FPI_2_ designed to match *λ*_ref_R_, which was 5 nm higher than that required for matching *λ*_ref_G_, resulting in values of 112, 115, 117, 118, and 119 nm, respectively. Among them, the strongest resonance and highest FPF_2_ values in the red region were observed when *d*_2_ was 118 nm when Ag__upper_'s thickness was 35 nm, as shown in Figure 3b. Despite being optimized for the red region, the peak wavelengths of FPF_2_ were also in accordance with *λ*_ref_G_ and *λ*_ref_R_, with deviations of 1–2 nm. Figure 3c displays the transmission spectrum obtained from the FPI_1_ on the PET substrate, calculated as the product of initial transmittance ($T_{1}$) and FPF_1_. The peak wavelengths of the transmission spectrum formed at 407, 465, 550, and 684 nm, accordingly.

Finally, the total transmittance of the DFPI structure is determined by multiplying $T_{1}$ and Fabry–Perot factors of both FPI_1_ and FPI_2_, which is calculated as follows.

$$T_{total}=T_{1}\times {\mathrm{FP}F}_{1}\times {\mathrm{FP}F}_{2}$$

(13) $$=\frac{n_{Air}}{n_{PET}}\frac{{\left|t_{1}\right|}^{2}{\left|t_{2}\right|}^{2}}{\left[1+{R_{a}}^{2}-2R_{a}\;\cos\left({\Delta \varphi }_{\mathrm{FP}1}\right)\right]}\frac{{\left|t_{4}\right|}^{2}}{\left[1+{R_{b}}^{2}-2R_{b}\;\cos\left({\Delta \varphi }_{\mathrm{FP}2}\right)\right]}$$

By adding the FPI_2_ to FPI_1_, fine‐tuning is achieved by effectively weighting and shifting the resonance peak positions. The overall transmittance spectrum as a function of Ag__upper_ thickness is shown in Figure 3d. As the Ag layer thickness increases from 20 to 40 nm, the peak wavelengths shift from 462, 536, and 634 nm to 465, 540, and 639 nm, respectively. Meanwhile, the transmittance at the red peak rises from 44.57% to 60.22%, whereas that of the blue and green peaks decreases from 64.82% to 24.09%, and 79.61%–42.69%, respectively. Furthermore, to compensate for this imbalance of light intensity in R, G, and B spectral regions, the thickness of Ag__upper_ was set to 35 nm, ensuring optimal transmittance in the red region.

Finally, the thickness of *d*_2_ was adjusted to ensure that the peak wavelengths in the DFPI transmittance spectrum aligned with *λ*_ref_B_, *λ*_ref_G_ and *λ*_ref_R_. When the FPI_2_ layers above the FPI_1_ were configured as 122 nm of WO_3_, 35 nm of Ag, and 60 nm of WO_3_, the resulting transmission peaks at 466 nm, 541 nm, and 643 nm, respectively. The corresponding transmittance intensities were 31.81% in blue, 52.13% in green, and 65.91% in red, fulfilling the design requirement for high transmittance in the red region. Figure 3e illustrates the calculation of transmission of light through the overall DFPI structure, expressed as a sequential product of individual optical terms. Also, the electric field profiles at each target wavelengths (*λ*_ref_B_, *λ*_ref_G_ and *λ*_ref_R_) were analyzed, as presented in Figure 3f. The results demonstrate that the resonance order of FPI_1_ were *m* = 6, 5, and 4 for B, G, and R, respectively, and the introduction of FPI_2_ enhanced the electric field intensity in the red region, which is consistent with the transmission spectrum shown in Figure 3e. These results confirm that the DFPI structure enables the independent stacking of two functional FPIs, allowing accurate control over spectral alignment and color balance in the visible region.

By combining a thick FPI for multiple resonance generation with a thinner FPI for spectral fine‐tuning, the DFPI introduces functional decoupling within the multilayer system. This strategy increases design flexibility and enables simultaneous control of spectral alignment and intensity redistribution beyond the limitations of a conventional single FPI.

### 2.3 Bi‐Directional Neural Network Assisted DFPI Design

While this analytical formulation provides a physical framework defining the DFPI design space, efficiently exploring this multi‐dimensional space to identify optimal structural configurations remains challenging. Conventional optical design techniques, such as electromagnetic simulations and transfer matrix methods, often rely on iterative parameter sweeps and are computationally expensive. Thus, data‐driven approaches have gained significant attention for their ability to efficiently extract information from the large datasets and predict optimal structural configurations. Thus, an artificial neural network (ANN) was employed to directly model the nonlinear relationship between structural parameters and their corresponding optical responses.

First, a forward neural network (FNN) was constructed to predict the transmission spectrum from a given set of structural parameters. The model adopts a fully connected layer (FCL) architecture, in which each neuron in a layer is connected to every neuron in the next layer. This architecture allows the network to model complex, high‐dimensional and nonlinear mappings between geometric parameters and their corresponding spectral characteristics.

A FNN model was constructed to predict the peak positions and transmittance values of blue, green, and red regions in the transmittance spectrum, based on the thicknesses of the multilayer DFPI structure. As illustrated in Figure 4a, the input parameters consist of five thicknesses (*d*_1_ to *d*_5_), corresponding to alternating metal and dielectric layers in DFPI structure on a PET substrate. The passivation layers were fixed at 60 nm on the top and 50 nm on the bottom, while the input parameters were defined as follows: metal layers *d*_1_, *d*_3_, and *d*_5_ ranged from 15 to 35 nm, 5–20 nm, and 20–45 nm, respectively, each in 5 nm increments. The dielectric layer *d*_2_ was varied in 10 nm steps from 580 to 760 nm to allow fourth or fifth order resonance modes in the R, G, and B regions. The fine tuning layer *d*_4_ ranged from 100 to 140 nm in 5 nm increments. In total, 20,520 data samples were generated to train the FNN model, and the distribution of each input parameter is shown in Figure 4b.

**FIGURE 4:**
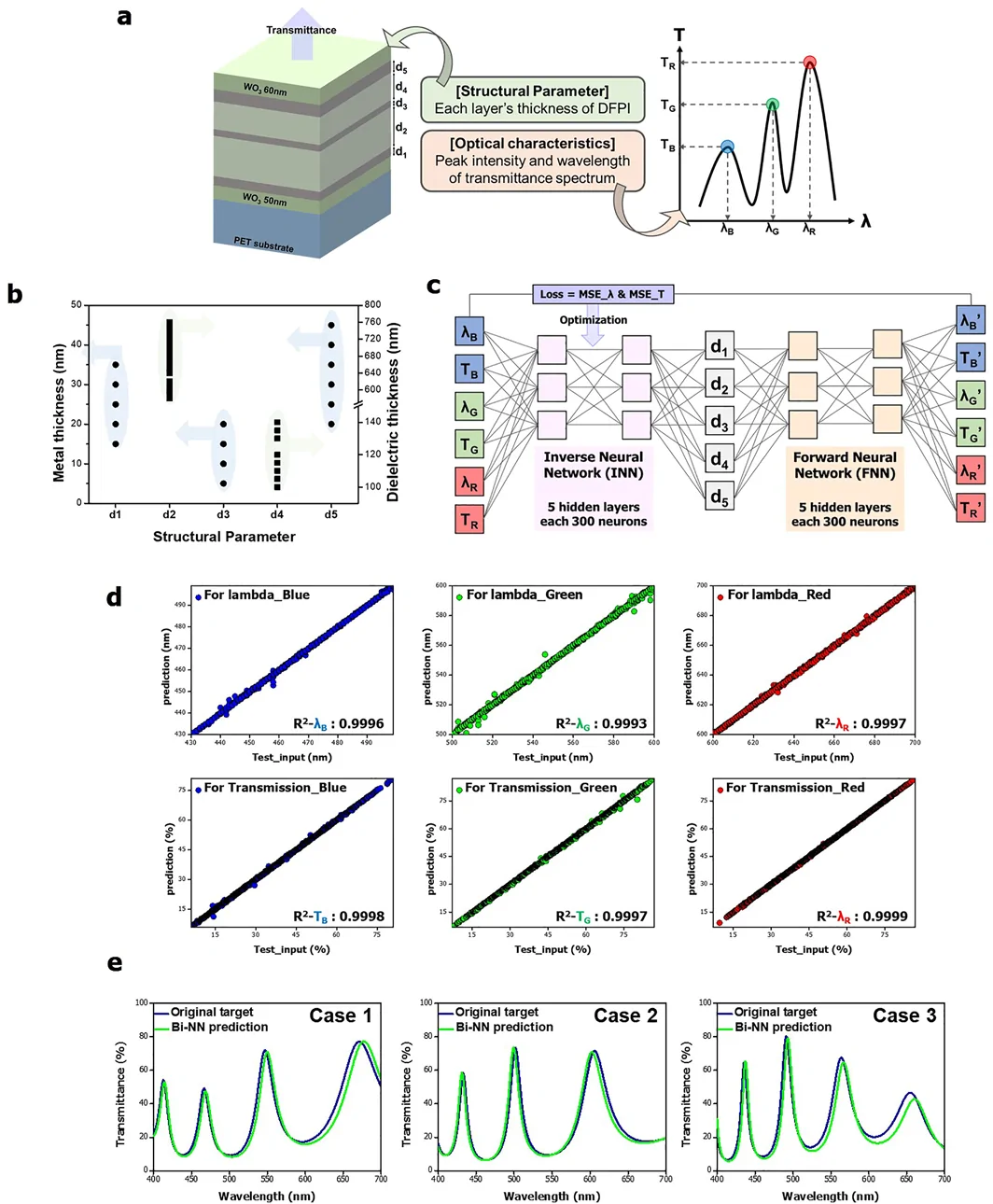
Bi‐directional neural network (Bi‐NN) based design for the optical DFPI structure. (a) The relationship between the five structural parameters (layer thicknesses) and six optical characteristics (peak wavelengths and transmittance values in the R, G, and B regions). (b) Distribution of structural parameters used to construct the learning dataset. (c) Schematic diagram of the Bi‐NN architecture composed of INN and pre‐trained FNN. (d) Scatter plots comparing Bi‐NN predictions with target test data for six optical characteristics. (e) Differences between the target and predicted values for three randomly selected samples obtained from the optimized Bi‐NN.

The output of the FNN consisted of six optical parameters: the peak wavelengths (*λ*_B_, *λ*_G_, *λ*_R_) and corresponding transmittance values (*T*_B_, *T*_G_, *T*_R_) in the transmittance spectrum. The schematic of the designed FNN model is illustrated in the yellow region of Figure 4c. The dataset was randomly divided into 85% for training, 7.5% for testing, and 7.5% for validation. All input data were normalized to the range of 0–1 to prevent scale related distortion during training. The ReLU (Rectified Linear Unit) activation function was applied, and the loss function Mean Square Error (MSE) was decomposed into two components: MSE_λ_ for the wavelengths predictions and MSE_T_ for the transmittance values. These two losses were simultaneously optimized during training. Each model was trained for 1000 epochs, and the number of hidden layers and neurons per layer were adjusted to minimize the total loss. As a result, the optimal model, consisting of five hidden layers with 300 neurons each, achieved minimum errors of MSE_λ_ 2.960 and MSE_T_ 1.249.

The trained FNN enables rapid prediction of optical responses from structural parameters without additional optimization process. To utilize this capability for practical design purposes, an inverse neural network (INN) was introduced to determine structural configurations that yield the desired optical responses. The conceptual schematic of INN is shown in the pink region of Figure 4c. Unlike the forward prediction, the inverse design problem is more complex because multiple structural designs can produce similar optical responses. This one‐to‐many relationship, known as the non‐uniqueness problem, often causes instability during training in conventional multi‐layer perceptron based INNs. Overcoming this limitation is critical for achieving reliable inverse design in multilayer optical systems.

To address this non‐uniqueness problem, a bi‐directional neural network (Bi‐NN) model was constructed by connecting a pre‐trained FNN to the output of an INN. A desired optical property *R* was first fed into the INN, and the predicted structural parameters were then passed through the FNN to generate the reconstructed optical output *R′*. The Bi‐NN was trained to minimize the discrepancy between *R* and *R′*, thereby guiding the inverse process to converge toward consistent and physically reliable solutions. The pre‐trained FNN serves as a supervisory signal, ensuring that inverse prediction corresponds to valid forward mappings. This indirect training strategy allows the model to overcome non‐uniqueness issues without requiring an explicit one‐to‐one correspondence.

The Bi‐NN model was optimized by changing the hyper‐parameters of the INN while keeping the pre‐trained FNN fixed at 300 neurons in five hidden layers. (Detailed information on optimization of each model is provided in Supporting Information S1: S10). Each configuration was trained for 1000 epochs and the minimum loss was obtained when INN also consisted of five hidden layers with 300 neurons per layer. The optimized Bi‐NN model was analyzed using 1539 test data that were not included in the training dataset. Figure 4d shows scatter plots comparing the original target values (*λ*_B_, *T*_B_, *λ*_G_, *T*_G_, *λ*_R_, *T*_R_) with corresponding Bi‐NN predictions (*λ*_B_′, *T*_B_′, *λ*_G_′, *T*_G_′, *λ*_R_′, *T*_R_′). All predicted parameters exhibited excellent agreement with the original dataset, with coefficient of determination (*R* ^2^) values exceeding 0.99, indicating high model accuracy and consistency.

To evaluate the performance of the trained Bi‐NN model, three additional target sets excluded from both the training and test datasets were used as inputs. The corresponding comparisons between the original target spectra and Bi‐NN predicted results, which include both reconstructed transmittance spectra are presented in Figure 4e. The predicted thickness of each layer deviated by less than 4 nm from their respective target values. Consequently, the predicted transmittance spectra exhibited excellent agreement with the original target spectra.

To further assess the practical performance of the model, the Bi‐NN was tested using target optical inputs of 466 nm, 28%, 541 nm, 45%, 643 nm, 60%, which were not included in the training dataset. The corresponding predicted optical outputs were 468.09 nm, 31.84%, 544.23 nm, 52.59%, 646.15 nm, 60.62%, with the predicted structural layer thicknesses of 27.80, 639.80, 9.92, 122.32, 36.82 nm, which are in close agreement with the reference design values of 25, 636, 10, 122, 35 nm. Both the optical outputs and the corresponding structural parameters exhibited excellent consistency with analytical formulation, confirming the reliability and accuracy of the proposed Bi‐NN for inverse structural prediction. This result demonstrates that the Bi‐NN framework can serve as an efficient complementary tool to analytical and simulation based approaches for stable inverse design under multi constraint conditions.

### 2.4 Experimental Verification and Optical Performance Analysis

The transmission spectrum of the DFPI structure, designed through both Bi‐NN and analytical formulation, and validated by experimental results is shown in Figure 5a. The DFPI optical film was fabricated on a 10 cm by 10 cm PET substrate using a thermal vapor deposition process. A photograph of the fabricated film is also included.

**FIGURE 5:**
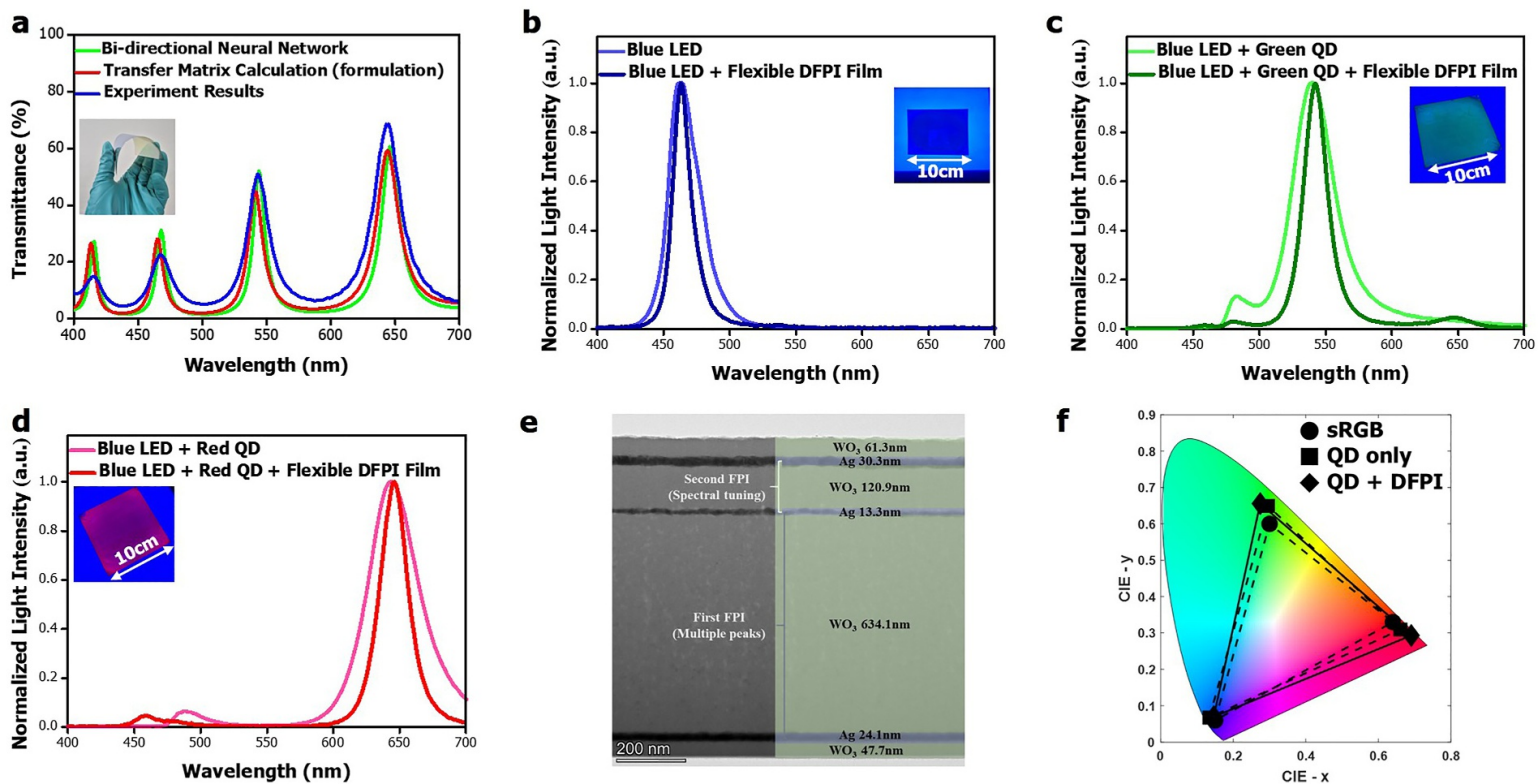
Experimental verification of the color purity enhancement by the fabricated flexible DFPI film. (a) Comparison of transmission spectra for the DFPI film design by analytical formulation, Bi‐NN, and actual experimental result. (b–d) Normalized FWHM comparison of emission spectra with and without the DFPI film applied to (b) blue LED, (c) green QD, and (d) red QD, respectively. (e) Transmission electron microscope (TEM) image of the flexible DFPI film captured at a magnification of 34,000x. (f) CIE‐1931 color coordinate comparison among the fabricated DFPI film, the reference light source, and the standard sRGB gamut.

When analyzing the transmittance spectrum of the fabricated film, the region B, G, and R have the peak wavelengths and corresponding values of 22.54% at 467 nm, 50.96% at 543 nm, and 63.80% at 643 nm, respectively. In comparison, the DFPI structure designed through analytical formulation showed slightly shifted peaks at 466, 541, and 643 nm, with transmittances of 27.74%, 44.84%, and 59.29%, respectively. The Bi‐NN design yielded peak wavelengths of 468, 544, and 646 nm, showing transmittances of 31.32%, 52.02%, and 60.65% for the respective color regions. Overall, the experimental results are in close agreement with the designed spectra, supporting the validity of both the analytical and Bi‐NN based approaches.

The fabricated DFPI film was placed on the reference light source to investigate for changes in FWHM of its emission spectrum. The emission spectra of three primary colors before and after the application of the DFPI film were normalized for comparison. The normalized spectra of B, G, and R are shown in Figure 5b–d, respectively. The application of the DFPI film led to a 41.99% reduction in FWHM for the blue LED, narrowing 27.36–15.87 nm. For the green and red QD films, the FWHM decreased by 42.82% and 44.46%, reducing from 36.57 to 20.91 nm, and from 44.26 to 24.58 nm, respectively.

To clarify the discrepancy between the measured transmittance and the designed spectrum, the cross‐sectional structure of the fabricated film was analyzed using a transmission electron microscope (TEM). Figure 5e shows the TEM image of a cross‐sectional TEM image captured at a magnification of 34,000x. The WO_3_ layers were deposited with thicknesses of 47.7, 634.1, 120.9, and 61.3 nm from the PET substrate, showing an average deviation of approximately 1.7 nm from the analytical design. For the Ag layers, the measured thicknesses were 24.1, 13.3, and 30.3 nm from the bottom, which also slightly differ from the original design. The observed variations in transmittance characteristics are considered to originate from surface roughness and fabrication imperfections.

To evaluate the effect of the DFPI film on color purity, the emission spectra before and after applying the DFPI film were converted into CIE‐1931 color coordinates and are shown in Figure 5f. For comparison, the standard sRGB gamut, the reference light source, and the color coordinates of the R, G, and B after applying the DFPI film are simultaneously plotted. The CIE‐xyz color coordinates of the reference light source were (0.13434, 0.06763), (0.29555, 0.64923), and (0.66088, 0.30953) for blue, green, and red, respectively. After applying the DFPI film, the corresponding color coordinates shifted toward the edge of the CIE‐xyz color space to (0.14623, 0.06978), (0.27429, 0.65573), and (0.69061, 0.29319), respectively. Consequently, the color gamut area increased by 9.14% compared to the reference light and by 30.15% relative to the sRGB color gamut. These results confirm that the DFPI structure effectively narrows the spectral FWHM and enhances color purity, as demonstrated through analytical formulation, neural network based design, and experimental validation.

To assess the flexibility of the DFPI film on a PET substrate, a bending test was conducted at a fixed bending radius of 30 mm, increasing the number of cycles in increments of 10,000 times. As shown in Figure 6a,b, the transmission peak wavelengths exhibited only minor shifts of 2.0, 3.5, and 2.5 nm for B, G, and R regions, respectively, relative to their reference wavelengths. The FWHM values of the transmitted spectra measured from the reference light passing through the DFPI film, also showed minimal deviations of 1.51 nm for blue, 1.40 nm for green, and 0.36 nm for red compared to unbent condition. These results indicate that the DFPI film retains its color purity enhancement capability throughout the bending test.

**FIGURE 6:**
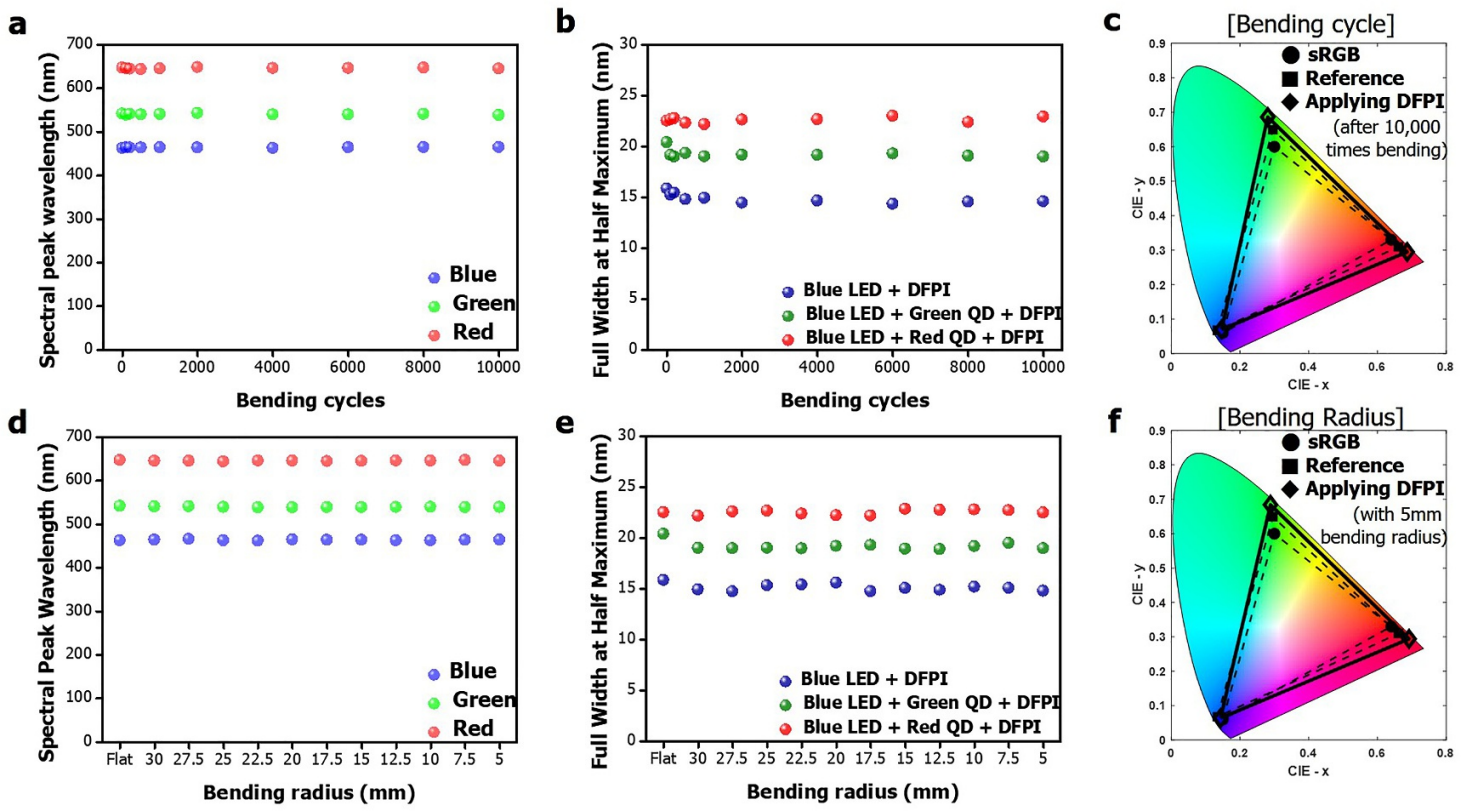
Evaluation of the flexibility characteristics of the DFPI film fabricated on a PET substrate. (a) Shifts in the R, G, and B peak positions of the DFPI film as a function of the bending cycle. (b) Variation of spectral FWHM of the DFPI applied to the reference light source with a bending cycle. (c) CIE‐1931 color coordinates comparing the DFPI film after 10,000 bending cycles with the reference light and the sRGB gamut. (d) Spectral peak shifts as a function of bending radius. (e) Change in spectral FWHM of the DFPI film under different bending radius. (f) Comparison of the CIE‐1931 color coordinates after a bending test with 5 mm radius with reference light and the sRGB gamut.

Figure 6c presents a comparison of CIE‐1931 color coordinates for three cases: the reference light source, the DFPI films after 10,000 bending cycles placed on top of the reference light, and standard sRGB gamut. The color gamut increased by 13.22% and 35.02% compared to reference light and sRGB, respectively, confirming that the DFPI film effectively maintains its color purity enhancement effect even after repeated bending.

A bending test with varying radius was conducted to further evaluate the flexibility of the DFPI film. Starting from the flat state, the bending radius was sequentially reduced in 2.5 mm steps down to 5 mm, with 1000 bending cycles applied at each step. Figure 6d,e present the shifts in the R, G, and B transmission peak wavelengths and the corresponding FWHM variations, measured under the same conditions as in Figure 6b. The spectral peak wavelengths showed a maximum shift of approximately 3–3.5 nm from their reference wavelengths. The corresponding FWHM changes were 1.13 nm for blue, 1.51 nm for green, and 0.36 nm for red.

As shown in Figure 6f, the CIE‐1931 color coordinates measured after the bending test indicate a color gamut increase of 14.85% and 36.96% compared with the reference light and sRGB gamut, even at a 5 mm bending radius. These results confirm that the fabricated DFPI film maintains stable spectral characteristics and color purity enhancement performance under mechanical deformation.

## 3 Methods

The transmittance and Fabry–Perot factor of DFPI were calculated using MATLAB (MathWorks) and FDTD solutions (Finite‐Difference Time‐Domain method; Lumerical Co. BC, Canada). The transfer matrix method and optical functions were implemented in MATLAB. The analytical formulation and electromagnetic simulations were conducted under standard thin film conditions, assuming planar multilayer geometry, linear, homogeneous and isotropic materials, and normal plane wave incidence. The electric field distribution of the DFPI was exported and visualized as an image using MATLAB and FDTD solutions.

A PET (polyethylene terephthalate) substrate (10 cm × 10 cm) was cleaned in an ultrasonic bath with isopropyl alcohol, followed by UV ozone treatment for 20 min. The refractive indices of silver (Ag) and tungsten trioxide (WO_3_) were measured using a spectroscopic ellipsometer (M‐2000D; J.A.Woollam., NE, USA). Ag (3–5 nm granules, 4N; Tasco Co., Anyang, Korea) and WO_3_ (1‐4 mmpcs, 4N; Tasco Co., Anyang, Korea) were deposited alternatively by thermal evaporation under a vacuum pressure below 5 × 10^−5^ torr without vacuum break. The deposition rates for Ag and WO_3_ were 1 Å/s and 2 Å/s, respectively.

The transmittance spectra of fabricated DFPI films were measured using a UV–Vis spectrometer (LAMBDA 365; PerkinElmer, MA, USA). The reference light source consisted of a blue LED and green, red QD films (10 cm × 10 cm). The transmitted light intensity was measured using a Black‐Comet UV–Vis spectrometer and SpectraWiz‐Shortcut software (Stellanet Inc., FL, USA). The cross‐sectional morphology of deposited DFPI film was captured using a transmission electron microscope (Themix Z TEM; Thermo Fisher scientific, MA, USA). The bending test was conducted using a twist bending system (ST‐TBJS‐081060; ST1, Incheon, Korea).

The neural network‐based inverse design of DFPI was implemented using Google Colaboratory with Tensorflow. The training dataset was generated through MATLAB simulations. All datasets were normalized from 0 to 1 using Scikit‐Learn to improve training stability and convergence. The Bi‐directional neural network was trained for 1000 epochs using the Adam optimizer (learning rate = 0.0001), with ReLU activation and mean square error (MSE) loss functions. Python 3.11 was used with Tensorflow and Scikit‐Learn for model development and data‐processing. The local computational environment included the Window 10 Education 64‐bit OS, an Intel Core i7‐11700 KF CPU, 32 GB RAM, and an NVIDIA GeForce GTX 1650 GPU.

## 4 Discussion

An optimized DFPI structure was proposed for integration with quantum dot color conversion films. When applied to a reference light source, the DFPI significantly reduced the FWHM compared to QD only devices, resulting in enhanced color purity. The optical performance of the multilayer thin film structure could be further enhanced by substituting metallic layers with low‐loss dielectric mirrors, such as DBR to minimize absorption losses. Although this approach may increase structural complexity and fabrication challenges, DBR integration represents a promising extension for maximizing color purity and spectral selectivity in future high performance implementations. The present DFPI functions as a passive optical component, it could be extended to active emissive devices by incorporating light emitting materials. In addition, to improve process compatibility, DFPI can be easily fabricated by various conformal evaporation techniques or wet‐coating processes. The DFPI architecture also offers improved tolerance to minor spectral variations and fabrication induced variations, thereby enhancing its applicability under practical display operating conditions. Furthermore, the proposed optical structure with high color purity is expected to enhance color reproduction even in form‐free or flexible configurations, broadening its applicability to diverse optical fields such as wearable displays and optical sensors [30].

To facilitate efficient DFPI structure design, a Bi‐NN was employed. The current model based on a simple FCL architecture ensures reliable accuracy, but it still requires large training datasets and offers limited design efficiency. Future research could adopt more compact architectures or data‐efficient learning strategies. Ultimately, the neural network methodology demonstrated in this work is anticipated to evolve into a next generation optical design approach, enabling the prediction and optimization of more complex photonic structures beyond the DFPI system [31].

## 5 Conclusion

A DFPI structure was developed to enhance the color purity through precise spectral control. By introducing an additional Fabry–Perot cavity to a conventional single FPI configuration, simultaneous alignment of the R, G, and B transmission peaks was achieved while enabling independent control of spectral redistribution and intensity balance. The total transmittance was analytically modeled as the product of individual Fabry–Perot factors, providing a physically intuitive framework for structural design. Furthermore, a data‐driven Bi‐NN was applied to optimize the inverse design process, enabling efficient prediction of structural parameters while addressing the non‐uniqueness problem inherent in multilayer optical systems.

The optimized DFPI film was fabricated on a PET substrate via thermal deposition, demonstrating high flexibility and optical stability. When applied to blue LED and QD color conversion layers, the film exhibited a 9.14% increase in color gamut relative to QD only devices and a 30.15% enhancement compared with the sRGB gamut. Mechanical bending tests confirmed the structural robustness and consistent spectral characteristics under deformation. These results establish an optical and AI design paradigm that bridges analytical modeling and data‐driven optimization for future high performance photonic applications.

## Author Contributions

K.S.S. and Y.S.D. conceived the research concept and overall design. K.S.S. established the analytical formulation of the optical model and experimental characterization. K.S.S. and K.W.J. performed the neural network computations and developed the bi‐directional neural network for inverse design. Y.S.D. supervised the entire project and secured funding. The manuscript was mainly written by K.S.S. with contributions of all authors.

## Funding

This work was supported by Basic Science Research Programs through the National Research Foundation of Korea (NRF), Ministry of Education (Grant NRF‐2022R1I1A3070928), the Basic Science Research Program through the National Research Foundation of Korea funded by Ministry of Science and ICT of Korea (No. RS‐2025‐02217276), and the Alchemist Project grant funded by Korea Planning & Evaluation Institute of Industrial Technology (KEIT) & the Korea Government (MOTIE) (Project Number: 1415185027, 20019169).

## Conflicts of Interest

The authors declare no conflicts of interest.

## Supporting information


Supporting Information S1

## Data Availability

The data that supports the findings of this study are available in the supplementary material of this article.
