# Supplementary material for: Flexible Dual Fabry–Perot Filters Enabling Color Balanced Narrowband Emission Designed by Physics Guided Neural Inverse Optimization
Source: Nanophotonics. 2026 May 13;15(10):e70135. doi: 10.1002/nap2.70135 (PMC13642490; doi:10.1002/nap2.70135)
Supplement: Supplementary file 1 [file NAP2-15-e70135-s001.docx]

Supplementary Information

**Flexible dual Fabry-Perot filters enabling color balanced narrowband emission designed by physics guided neural inverse optimization.**

Keun Soo Shin, Ki Won Jeong, and Yun Seon Do*

School of Electronic and Electrical Engineering, Kyungpook National University, Daegu 41566, Republic of Korea.

[*yuns.do@knu.ac.kr](mailto:*yuns.do@knu.ac.kr)

**S1. Refractive Indices of Ag, WO_3_, and PET films within the visible range.**

**Figure S1:** Complex refractive index graphs of Ag, WO_3_, and PET utilized in DFPI structure design.

**S2. Measured spectral intensities of R, G, and B in the reference light.**

Green and red quantum dot (QD) films, which act as color conversion layers, were placed on the Blue LED. The light intensities of each R, G, and B region were measured with a UV-vis spectrometer. As a result, the emission peak wavelengths of 61,392 W/m^2^ in blue, 36,144 W/m^2^ in green, 25,694 W/m^2^ in red. Based on this, DFPI was designed to achieve higher transmission in the red region of the transmission spectrum.

**Figure S2:** The measured emission spectra of blue LED, green and red QD films, with sprectral peak wavelengths at 466, 541, and 643 nm.
 respectively.

**S3. Transfer Matrix Methods (TMM) for multilayer thin films.**

**Figure S3:** The diagram for applying TMM to multilayer thin films.

**① Optical admittance**

$$Y= \frac{H}{E}=n\sqrt{\frac{\varepsilon_{0}}{\mu_{0}}}=n\times2.654\times{10}^{-3}$$

**② Boundary condition for the electromagnetic field (at boundary 1)**

$$E_{1}= {E_{01}}^{+}+ {E_{01}}^{-}= {E_{11}}^{+}+ {E_{11}}^{-}$$

$$H_{1}= {H_{01}}^{+}- {H_{01}}^{-}= {H_{11}}^{+}- {H_{11}}^{-}$$

$$H_{1}= {Y_{0}(E_{01}}^{+}- {E_{01}}^{-})= {{Y_{1}(E}_{11}}^{+}- {E_{11}}^{-})$$

Using $H=EY,$

**③ Phase Shift**

with $\phi_{1}= \tilde{n_{1}}k_{0}d_{1}$

$${E_{11}}^{-}= {E_{12}}^{-}e^{-j\phi_{1}}$$

${E_{11}}^{+}= {E_{12}}^{+}e^{j\phi_{1}}$,

${E_{12}}^{+}= {E_{11}}^{+}e^{-j\phi_{1}}$,

$${H_{11}}^{-}= {H_{12}}^{-}e^{-j\phi_{1}}$$

${H_{11}}^{+}= {H_{12}}^{+}e^{j\phi_{1}}$,

${H_{12}}^{+}= {H_{11}}^{+}e^{-j\phi_{1}}$,

$$E_{1}= {E_{11}}^{+}+ {E_{11}}^{-}= {E_{12}}^{+}e^{j\phi_{1}}+{E_{12}}^{-}e^{-j\phi_{1}}$$

$$H_{1}=Y_{1}({E_{11}}^{+}- {H_{11}}^{-})=Y_{1}({E_{12}}^{+}e^{j\phi_{1}}-{E_{12}}^{-}e^{-j\phi_{1}})$$

**④ Electromagnetic field at the boundary 2**

$$E_{2}= {E_{12}}^{+}+ {E_{12}}^{-}= {E_{22}}^{+}+ {E_{22}}^{-}= \frac{1}{Y_{1}}\left( {H_{12}}^{+}+ {H_{12}}^{-} \right)= \frac{1}{Y_{2}}({H_{22}}^{+}+ {H_{22}}^{-})$$

$$H_{2}= {H_{12}}^{+}- {H_{12}}^{-}= {H_{22}}^{+}- {H_{22}}^{-}= Y_{1}\left( {E_{12}}^{+}- {E_{12}}^{-} \right)= Y_{2}({E_{22}}^{+}- {E_{22}}^{-})$$

${H_{12}}^{-}= \frac{1}{2}\left( Y_{1}E_{2}- H_{2} \right)$.

${H_{12}}^{+}= \frac{1}{2}(Y_{1}E_{2}+ H_{2})$,

${E_{12}}^{-}= \frac{1}{2}(E_{2} - \frac{H_{2}}{Y_{1}})$,

Then ${E_{12}}^{+}= \frac{1}{2}(E_{2} + \frac{H_{2}}{Y_{1}})$,

ⅱ) ${E_{11}}^{-}= {E_{12}}^{-}e^{-j\phi_{1}}=\frac{1}{2}(E_{2} - \frac{H_{2}}{Y_{1}})e^{-j\phi_{1}}$

ⅰ) ${E_{11}}^{+}= {E_{12}}^{+}e^{j\phi_{1}}=\frac{1}{2}(E_{2} + \frac{H_{2}}{Y_{1}})e^{j\phi_{1}}$

ⅳ) ${H_{11}}^{-}= {H_{12}}^{-}e^{-j\phi_{1}}=\frac{1}{2}(Y_{1}E_{2}- H_{2})e^{-j\phi_{1}}$

ⅲ) ${H_{11}}^{+}= {H_{12}}^{+}e^{j\phi_{1}}=\frac{1}{2}(Y_{1}E_{2}+ H_{2})e^{j\phi_{1}}$

**⑤ Transfer Matrix**

$E_{1}= {E_{11}}^{+}+ {E_{11}}^{-}= E_{2}\left( \frac{e^{j\phi_{1}}+e^{-j\phi_{1}}}{2} \right)+ H_{2}\left( \frac{e^{j\phi_{1}}-e^{-j\phi_{1}}}{2Y_{1}} \right)= E_{2}\cos\left( \phi_{1} \right)+ H_{2}\left( \frac{jsin\left( \phi_{1} \right)}{Y_{1}} \right)$,

For E-field,

For H-field,

$H_{1}= {H_{11}}^{+}- {H_{11}}^{-}= E_{2}Y_{1}\left( \frac{e^{j\phi_{1}}-e^{-j\phi_{1}}}{2} \right)+ H_{2}\left( \frac{e^{j\phi_{1}}+e^{-j\phi_{1}}}{2} \right)= E_{2}(jY_{1}\sin\left( \phi_{1} \right))+ H_{2}cos(\phi_{1})$.

$\therefore$ $\left[ \begin{aligned} E_{1} \\ H_{1} \end{aligned} \right]= \left[ \begin{matrix} \cos(\phi_{1}) & \frac{jsin(\phi_{1})}{Y_{1}} \\ jY_{1}\sin(\phi_{1}) & \cos(\phi_{1}) \end{matrix} \right]\left[ \begin{aligned} E_{2} \\ H_{2} \end{aligned} \right]$

For generalized version,

$$\left[ \begin{aligned} E_{1} \\ H_{1} \end{aligned} \right]= \left[ \begin{matrix} \cos(\phi_{1}) & \frac{jsin(\phi_{1})}{Y_{1}} \\ jY_{1}\sin(\phi_{1}) & \cos(\phi_{1}) \end{matrix} \right]\left[ \begin{matrix} \cos(\phi_{2}) & \frac{jsin(\phi_{2})}{Y_{2}} \\ jY_{2}\sin(\phi_{2}) & \cos(\phi_{2}) \end{matrix} \right]\cdot\cdot\cdot\left[ \begin{matrix} \cos(\phi_{n-1}) & \frac{jsin(\phi_{n-1})}{Y_{n-1}} \\ jY_{n-1}\sin(\phi_{n-1}) & \cos(\phi_{n-1}) \end{matrix} \right]\left[ \begin{aligned} E_{n} \\ H_{n} \end{aligned} \right]$$

$$\left[ \begin{aligned} {\boldsymbol{E}_{\boldsymbol{1}}}/{\boldsymbol{E}_{\boldsymbol{n}}} \\ {\boldsymbol{H}_{\boldsymbol{1}}}/{\boldsymbol{E}_{\boldsymbol{n}}} \end{aligned} \right]\boldsymbol{=}\left[ \begin{aligned} \boldsymbol{B} \\ \boldsymbol{C} \end{aligned} \right]\boldsymbol{=}\left[ \boldsymbol{M} \right]\left[ \begin{aligned} \boldsymbol{1} \\ \boldsymbol{Y}_{\boldsymbol{n}} \end{aligned} \right]$$

$$\left[ \begin{aligned} E_{1} \\ H_{1} \end{aligned} \right]= \left[ M \right]\left[ \begin{aligned} E_{n} \\ H_{n} \end{aligned} \right]$$

**⑥ Reflectance**

At the boundary 1, $r_{01}= \frac{{E_{01}}^{-}}{{E_{01}}^{+}}$

$$E_{1}= {E_{01}}^{+}+ {E_{01}}^{-}= {E_{01}}^{+}(1+ r_{01})$$

$$H_{1}= {H_{01}}^{+}- {H_{01}}^{-}= {{{{Y_{0}(E}_{01}}^{+}- {E_{01}}^{-})= Y_{0}E}_{01}}^{+}(1- r_{01})$$

$$r_{01}= \frac{BY_{0}-C}{BY_{0} +C}$$

$$\frac{H_{1}}{E_{1}}= \frac{Y_{0}(1 - r_{01})}{1+ r_{01}}= \frac{C}{B}$$

so, Reflectance $\boldsymbol{R=}\frac{\boldsymbol{n}_{\boldsymbol{0}}}{\boldsymbol{n}_{\boldsymbol{0}}} \boldsymbol{\times}{\boldsymbol{|r}_{\boldsymbol{01}}\boldsymbol{|}}^{\boldsymbol{2}}\boldsymbol{=}{\boldsymbol{|}\frac{\boldsymbol{B}\boldsymbol{Y}_{\boldsymbol{0}}\boldsymbol{-C}}{\boldsymbol{B}\boldsymbol{Y}_{\boldsymbol{0}}\boldsymbol{+C}}\boldsymbol{|}}^{\boldsymbol{2}}$

**⑦ Transmittance**

At the boundary n, $t_{0n}= \frac{{E_{nn}}^{+}}{{E_{01}}^{+}}$

$$E_{1}= \frac{E_{1}}{E_{n}}E_{n}=BE_{n}=B{E_{nn}}^{+}= {E_{01}}^{+}+ {E_{01}}^{-}= {E_{01}}^{+}(1+ r_{01})$$

where ${E_{nn}}^{-}=0$

so, Transmittance $\boldsymbol{T=}\frac{\boldsymbol{n}_{\boldsymbol{n}}}{\boldsymbol{n}_{\boldsymbol{0}}} \boldsymbol{\times}{\boldsymbol{|t}_{\boldsymbol{0}\boldsymbol{n}}\boldsymbol{|}}^{\boldsymbol{2}}\boldsymbol{=}{\frac{\boldsymbol{n}_{\boldsymbol{n}}}{\boldsymbol{n}_{\boldsymbol{0}}} \boldsymbol{\times|}\frac{\boldsymbol{2}\boldsymbol{Y}_{\boldsymbol{0}}}{\boldsymbol{B}\boldsymbol{Y}_{\boldsymbol{0}}\boldsymbol{+C}}\boldsymbol{|}}^{\boldsymbol{2}}$

$$t_{0n}= \frac{1+ r_{01}}{B}= \frac{2Y_{0}}{BY_{0}+C}$$

**S4. Fabry-Perot factor in thin film optics.**

**Figure S4:** Conceptual diagram for deriving Fabry-Perot factor, assuming that external light enters in FPI interior.

where $R=\left| r_{1} \right|\left| r_{2} \right|e^{-\kappa k_{0}2d}$, $\Delta\phi_{FP}={- \phi}_{1} -\phi_{2} + 2nk_{0}d$

$$=Re^{-j\Delta\phi_{FP}}$$

$$=\left| r_{1} \right|\left| r_{2} \right|{e^{-\kappa k_{0}2d}e}^{-j({- \phi}_{1} -\phi_{2} + 2nk_{0}d)}$$

$$=\left| r_{1} \right|\left| r_{2} \right|{e^{j\phi_{1}}e^{j\phi_{2}}e}^{-jnk_{0}2d}e^{-\kappa k_{0}2d}$$

$$=\left| r_{1} \right|e^{j\phi_{1}}\left| r_{2} \right|e^{j\phi_{2}}e^{-j\tilde{n}k_{0}2d}$$

$$=\left| r_{1} \right|e^{j\phi_{1}}\left| r_{2} \right|e^{j\phi_{2}}e^{-j\tilde{n}k_{0}2d}$$

with$h= r_{1}r_{2}e^{-j\tilde{n}k_{0}2d}$

$$I_{1}= I_{0}\cdot t_{2}$$

$$I_{2}= I_{0}\cdot{(r}_{1}r_{2}e^{-j\tilde{n}k_{0}2d}{)\cdot t}_{2}$$

$$I_{3}= I_{0}\cdot{{(r}_{1}r_{2}e^{-j\tilde{n}k_{0}2d})}^{2}\cdot t_{2}$$

$I_{total}= I_{1}+ I_{2}+ I_{3}+ I_{4}+ \cdot$∙∙ $= I_{0}\frac{t_{2}}{1 -h},$ where $h= r_{1}r_{2}e^{-j\tilde{n}k_{0}2d}$

- Reflective Coefficients $r_{1}=|r_{1}|e^{{j\phi}_{1}}$_,_ $r_{2}=|r_{2}|e^{{j\phi}_{2}}$
- Initial condition (light amount) $I_{0}$
- Phase shift term : $e^{-jnkd}$

$I_{total}= I_{0}\frac{t_{2}}{1 -h}$ $= I_{0}\frac{t_{2}}{1 -Re^{-j\Delta\phi_{FP}}}$

For resonant condition,

$\Delta\phi_{FP}=2m\pi$ ($m$ is integer), $k_{0}= \frac{2\pi}{\lambda}$

$2nk_{0}d$ $=2m\pi$ ${+ \phi}_{1}+\phi_{2}$

$$d=(\frac{\phi_{1}+\phi_{2}+ 2m\pi}{4n\pi})\cdot\lambda$$

So, Fabry-Perot factor $f_{FP}\left( \lambda\right)$

$$\boldsymbol{f}_{\boldsymbol{FP}}\left( \boldsymbol{\lambda} \right)\boldsymbol{=}\frac{{\boldsymbol{|I}_{\boldsymbol{total}}\boldsymbol{|}}^{\boldsymbol{2}}}{{\boldsymbol{|I}_{\boldsymbol{0}}\boldsymbol{|}}^{\boldsymbol{2}}}\boldsymbol{=}\frac{{\boldsymbol{|t}_{\boldsymbol{2}}\boldsymbol{|}}^{\boldsymbol{2}}}{\boldsymbol{1+}\boldsymbol{R}^{\boldsymbol{2}}\boldsymbol{-2}\boldsymbol{Rcos(}\boldsymbol{\Delta}\boldsymbol{\phi}_{\boldsymbol{FP}}\boldsymbol{)}}$$

where $R=\left| r_{1} \right|\left| r_{2} \right|e^{-\kappa k_{0}2d}$, $\Delta\phi_{FP}={- \phi}_{1} -\phi_{2} + 2nk_{0}d$

$$={{|I}_{0}|}^{2}\frac{{{|t}_{2}|}^{2}}{1+ R^{2} -2Rcos(\Delta\phi_{FP})}$$

$$={{|I}_{0}|}^{2}\frac{{{|t}_{2}|}^{2}}{{\{\left( 1-Rcos\left( \Delta\phi_{FP} \right) \right) +jRsin(\Delta\phi_{FP})\}}^{2}}$$

$${{|I}_{total}|}^{2}={{|I}_{0}|}^{2}\frac{{{|t}_{2}|}^{2}}{|{1 -Re^{-j\Delta\phi_{FP}}|}^{2}}$$

**S5. Transmittance analysis with varying thickness of the upper WO_3_
layer in the DFPI.**

**Figure S5:** Fabry-Perot factor 1 (FPF_1_) spectra of FPI_1_ calculated using higher-order resonance modes designed to match λ_ref_B_ = 466 nm, λ_ref_G_ = 541 nm, and λ_ref_R_ = 643 nm.

**S6. Transmittance analysis with varying thickness of the upper WO_3_
layer in the DFPI.**

In addition to 35 nm Ag layer case, a parametric study was conducted by varying the Ag thickness from 20 nm to 40 nm while analyzing the transmittance dependence on the upper WO_3_ layer thickness (d_up_). The results showed that the transmittance in the red region consistently reached its maximum when d_up_ was 60 nm, regardless of the Ag layer thickness.

**Figure S6:** Transmittance characteristics according to changes in WO_3_ thickness (d_up_) for different Ag layer thickness.

**S7. Validation of the designed DFPI analytical model.**

The overall DFPI transmittance formulated as the product of individual Fabry-Perot factors (FPFs) was validated by comparison with the results obtained from the conventional Transfer Matrix Method (TMM) and optical simulation using the Finite Difference Time Domain (FDTD). The transmittance spectra from all three approaches showed excellent agreement, confirming the accuracy of the proposed analytical formulation.

**Figure S7:** Comparison of overall DFPI transmittance spectra obtained from the analytical formula, transfer matrix method (TMM), and optical simulation (finite different time domain, FDTD).

**S8. Quantitative comparison of color balance performance between a single Fabry–Perot cavity and the proposed DFPI structure.**

**Figure S8:** Comparison of PL spectra of the reference light source before and after applying a single cavity and designed DFPI configuration, illustrating enhanced RGB intensity balance.

**S9. Transmission variation of the DFPI as a function of cavity length.**

**Figure S9:** Simulated transmission spectra under ±5 nm cavity length variations for each cavity, demonstrating spectral stability within nanometer scale thickness variations.

**S10. Optimization of the Bi-directional neural network (Bi-NN) in theDFPI design.**

**Figure S10:** Optimization results of the FNN and Bi-NN training process, expressing the variation of the error function (MSE_λ_ and MSE_T_, and MSE_avg_).

We conducted an optimization process to find the minimum value of the error fuction (MSE) for the FNN and Bi-NN models. Learning was conducted a total 1,000 times while adjusting the hidden layer and the number of neurons of the model. As a result, both FNN and Bi-NN had a minimum MSE value when there five hidden layers and 300 neurons in each.

| **Layers**  **(neurons)** | **Forward neural network** | | | **Bi-directional neural network** | | |
| --- | --- | --- | --- | --- | --- | --- |
|  | **MSE_λ_** | **MSE_T_** | **MSE_avg_** | **MSE_λ_** | **MSE_T_** | **MSE_avg_** |
| **2 (20)** | 15.2621 | 6.1454 | 10.7053 | 6.3851 | 5.8942 | 6.1397 |
| **2 (30)** | 12.6744 | 5.0626 | 8.8685 | 6.1384 | 5.1143 | 5.6263 |
| **2 (50)** | 11.6151 | 4.3791 | 7.9971 | 5.3868 | 4.5586 | 4.9727 |
| **2 (100)** | 9.9916 | 3.3587 | 6.6752 | 5.0165 | 3.8880 | 4.4523 |
| **2 (200)** | 8.6229 | 2.7420 | 5.6825 | 4.6153 | 3.3272 | 3.9713 |
| **2 (300)** | 7.7922 | 2.3335 | 5.0629 | 4.8299 | 3.1908 | 4.0104 |
| **2 (400)** | 7.5042 | 2.1185 | 4.8114 | 4.2148 | 2.9932 | 3.6040 |
| **3 (20)** | 12.8629 | 5.8396 | 9.3513 | 9.8192 | 4.9813 | 7.4003 |
| **3 (30)** | 11.2232 | 4.7363 | 7.9796 | 5.4008 | 4.6182 | 5.0095 |
| **3 (50)** | 9.0999 | 3.4423 | 6.2711 | 4.8865 | 3.9211 | 4.4038 |
| **3 (100)** | 7.2611 | 2.6439 | 4.9525 | 4.4952 | 3.3802 | 3.9377 |
| **3 (200)** | 6.8333 | 2.0117 | 4.4225 | 4.4420 | 3.0891 | 3.7656 |
| **3 (300)** | 5.2951 | 1.9052 | 3.6002 | 4.3211 | 2.8303 | 3.5756 |
| **3 (400)** | 5.7452 | 1.4730 | 3.6091 | 3.8229 | 2.7149 | 3.2689 |
| **4 (20)** | 11.4077 | 4.7603 | 8.0840 | 5.8649 | 5.4076 | 5.6364 |
| **4 (30)** | 8.8918 | 4.0856 | 6.4887 | 6.0491 | 4.7055 | 5.3773 |
| **4 (50)** | 7.9476 | 3.2589 | 5.6033 | 4.5433 | 3.7838 | 4.1636 |
| **4 (100)** | 5.7459 | 2.3972 | 4.0716 | 4.3546 | 3.2299 | 3.7923 |
| **4 (200)** | 4.8791 | 1.7655 | 3.3223 | 4.4239 | 2.7573 | 3.5906 |
| **4 (300)** | 3.4386 | 1.2774 | 2.3580 | 4.0215 | 2.5934 | 3.3075 |
| **4 (400)** | 3.3536 | 1.5089 | 2.4313 | 4.1157 | 2.1843 | 3.150 |
| **5 (20)** | 11.8454 | 5.3054 | 8.5754 | 5.6818 | 5.2431 | 5.4624 |
| **5 (30)** | 9.8290 | 3.8489 | 6.8389 | 5.3103 | 4.2333 | 4.7718 |
| **5 (50)** | 7.6084 | 3.3055 | 5.4569 | 4.9125 | 3.4606 | 4.1865 |
| **5 (100)** | 4.9898 | 1.9091 | 3.4495 | 4.6748 | 3.2063 | 3.9405 |
| **5 (200)** | 3.5961 | 1.5788 | 2.5875 | 4.0346 | 2.8561 | 3.4454 |
| **5 (300)** | 2.960 | 1.2490 | 2.1045 | 4.1665 | 2.2954 | 3.2309 |
| **5 (400)** | 3.1986 | 1.2777 | 2.2382 | 4.3707 | 2.6042 | 3.4875 |
| **6 (20)** | 10.8805 | 5.2135 | 8.0470 | 9.4529 | 4.0308 | 6.7418 |
| **6 (30)** | 10.4219 | 4.2186 | 7.3203 | 5.0506 | 4.2711 | 4.6608 |
| **6 (50)** | 6.1062 | 2.9825 | 4.5443 | 4.6978 | 3.6521 | 4.1749 |
| **6 (100)** | 4.3967 | 2.1001 | 3.2484 | 5.0391 | 2.9629 | 4.0009 |
| **6 (200)** | 5.0491 | 2.0365 | 3.5428 | 8.5432 | 3.0757 | 5.8095 |
| **6 (300)** | 4.8850 | 1.9314 | 3.4082 | 4.1407 | 2.3572 | 3.2489 |
| **6 (400)** | 3.2241 | 1.4101 | 1.3171 | 4.6580 | 3.4032 | 4.0363 |
| **7 (20)** | 10.1666 | 5.1457 | 7.6562 | 5.3695 | 5.6095 | 5.4895 |
| **7 (30)** | 7.683 | 3.6747 | 5.6793 | 5.4609 | 4.3131 | 4.8871 |
| **7 (50)** | 6.8612 | 3.1257 | 4.9935 | 4.5111 | 3.4579 | 3.9845 |
| **7 (100)** | 4.1918 | 1.9890 | 3.0904 | 4.0385 | 2.9877 | 3.5131 |
| **7 (200)** | 3.1871 | 1.5678 | 2.3775 | 4.2385 | 2.5140 | 3.3763 |
| **7 (300)** | 3.7720 | 1.8987 | 2.8354 | 5.0428 | 2.8427 | 3.9427 |
| **7 (400)** | 3.7362 | 1.5532 | 2.6447 | 4.3642 | 2.7248 | 3.5555 |

**Table S1:** Summary of MSE values for each configuration of the FNN and Bi-NN models.

**S11. Prediction results of the optimized Bi-NN model.**

**Table S2:** Quantitive comparison between the target values and Bi-NN predicted results for three randomly

selected samples, corresponding to the data shown in Fig 4-e.
